# Body Integrity Identity Disorder

**DOI:** 10.1371/journal.pone.0034702

**Published:** 2012-04-13

**Authors:** Rianne M. Blom, Raoul C. Hennekam, Damiaan Denys

**Affiliations:** 1 Department of Psychiatry, Academic Medical Centre, University of Amsterdam, Amsterdam, The Netherlands; 2 Departments of Paediatrics and Translational Genetics, Academic Medical Centre, University of Amsterdam, Amsterdam, The Netherlands; 3 The Netherlands Institute for Neuroscience, an institute of the Royal Netherlands Academy of Arts and Sciences, Amsterdam, The Netherlands; The University of Melbourne, Australia

## Abstract

**Introduction:**

Body Integrity Identity Disorder (BIID) is a rare, infrequently studied and highly secretive condition in which there is a mismatch between the mental body image and the physical body. Subjects suffering from BIID have an intense desire to amputate a major limb or severe the spinal cord in order to become paralyzed. Aim of the study is to broaden the knowledge of BIID amongst medical professionals, by describing all who deal with BIID.

**Methods:**

Somatic, psychiatric and BIID characteristic data were collected from 54 BIID individuals using a detailed questionnaire. Subsequently, data of different subtypes of BIID (i.e. wish for amputation or paralyzation) were evaluated. Finally, disruption in work, social and family life due to BIID in subjects with and without amputation were compared.

**Results:**

Based on the subjects' reports we found that BIID has an onset in early childhood. The main rationale given for their desire for body modification is to feel complete or to feel satisfied inside. Somatic and severe psychiatric co-morbidity is unusual, but depressive symptoms and mood disorders can be present, possibly secondary to the enormous distress BIID puts upon a person. Amputation and paralyzation variant do not differ in any clinical variable. Surgery is found helpful in all subjects who underwent amputation and those subjects score significantly lower on a disability scale than BIID subjects without body modification.

**Conclusions:**

The amputation variant and paralyzation variant of BIID are to be considered as one of the same condition. Amputation of the healthy body part appears to result in remission of BIID and an impressive improvement of quality of life. Knowledge of and respect for the desires of BIID individuals are the first steps in providing care and may decrease the huge burden they experience.

## Introduction

Body Integrity Identity Disorder (BIID) is a rare condition in which persons typically report an intense desire either to be paralyzed or to have one or more of their healthy limbs to be amputated [1]–[3]. BIID is not a paraphilia [4] nor does the desire to amputate the limb reflect psychosis amputation [5]. Rather it is believed that BIID is an identity disorder [1]–[3], [6]. The main motivation for the preferred body modification is believed to be a mismatch between actual and perceived body schema [1], [7]. The symptoms of BIID parallel those in somatoparaphrenia, a syndrome occurring secondary to right parietal lobe damage by a cerebral tumor or stroke. This similarity, coupled with the early onset, suggests that BIID could be a congenital disorder [7]–[10].

Currently BIID is not included in the International Statistical Classification of Diseases 11 or the Diagnostic and Statistical Manual of Mental Disorders IV. As such this disorder is often not known to surgeons, neurologist and psychiatrists. To exasperate this issue, BIID individuals typically avoid healthcare and often act out their desires by pretending they are disabled or perform actual self-amputation [1], [11]–[14].

Previous studies on BIID almost exclusively focus on the desire for amputation [1], [15], [16]. However, on internet-based forums also people with a wish for a disability other than amputation describe that they recognize themselves as having BIID [17], [18]. Therefore, some researchers have proposed to broaden the intended use of BIID to refer to individuals with a persistent desire to acquire a physical disability [2], [3]. These other variants of BIID have not been investigated so far.

The present study aims to provide detailed phenomenology of BIID through the use of a detailed questionnaire given to a large group of BIID individuals. Since studies on BIID are limited our main goal is to broaden the knowledge of BIID in all healthcare professionals. This is done by describing all who deal with BIID and by determining whether BIID variants are significantly different.

## Methods

### Objectives

Main objective of the study is to provide detailed somatic, psychiatric, social and BIID characteristics of a large group of BIID individuals, in order to broaden the knowledge of BIID amongst all medical professionals. Secondary, objectives are to compare BIID variants on clinical measures and to compare disruption in work, social and family life due to BIID in amputated versus non-amputated subjects.

### Participants and procedures

Subjects who had identified themselves as having BIID (i.e. recognizing themselves in the following sentence: *“BIID is a term that covers several conditions in which people feel their body-image does not match with their body shape. When we use the term “BIID” or “BIID feelings” here we mean to indicate all these different forms of the condition. For example, some people would like to have their leg to be amputated under their knee, whereas others prefer to resemble someone who is paralysed.”)* were recruited between 24.12.2010 and 01.11.2011. Participants included (1) referrals from the psychiatry department of the Academic Medical Center Amsterdam (n = 6); (2) responders to research announcements distributed on BIID related websites (n = 42); (3) referrals from individuals who had already participated in the study (n = 7).

Since BIID is a highly secretive condition, all first communications were through the internet. Individuals which indicated to be interested were sent by e-mail full participant information by e-mail. After returning written consent, participants were invited to visit a secured website for the questionnaires. Five individuals preferred to visit the clinic and were seen in person. Fifty-eight subjects recognized themselves as having BIID and completed the survey. However, in 4 subjects the ‘not feeling complete in their own body’ was not the main motivation for body modification: one subjects' reason to modify his body was *to feel sexually aroused*, two subjects because of *the attention it draws* and for the last subject because *the process of modification* was the main focus of the desire. In order to generate a homogeneous sample, those 4 subjects were excluded from all analyses.

The questionnaire consisted of 6 parts totalling 112 questions, usually multiple choice, with space for additional comments or options. The BIID Phenomenology Questionnaire was build by the authors, included epidemiologic, medically directed, and specific BIID related questions, and included results from previous reports [1], [15], [16] (questionnaire available as Figure S1).

The Sheehan Disability Scale (SDS) is scale measuring functional impairment due to illness in work, family and social life [19].

To measure the severity of the BIID symptoms we adapted the Yale-Brown Obsessive-Compulsive Scale (Y-BOCS) [20], [21]. In 5-item scales (range 0–20) individuals were asked about the time they spent; the interference they experienced due to; the distress they had caused by; the resistance against; and the control they had over thoughts and activities of their BIID. Scores from 0–3 are considered indicative for subclinical BIID symptoms, 4–7 mild, 8–11 moderate, 12–15 severe, and 16–20 extreme (Y-BOCS questionnaire available as Figure S2).

The Mini-International Neuropsychiatric Interview Screen (MINI screen) is a self-rated, 25-item scale screening for the most common psychiatric disorders (i.e. depression, dysthymia, bi-polar disorder, panic disorder, social phobia, obsessive-compulsive disorder, post traumatic stress disorder, psychotic disorder, substance abuse, anorexia nervosa, bulimia nervosa, general anxiety disorder) [22].

The Beck Anxiety Inventory (BAI) is a self-rated, 21-item inventory measuring the severity of anxiety symptoms [23].

The Beck Depression Inventory (BDI): is a self-rated, 21-item inventory measuring the severity of depressive symptoms [24].

### Ethics

The study has been approved by the Medical Ethical Committee of the AMC-Amsterdam in accordance with the Declaration of Helsinki amended in Seoul in 2008.

### Statistical methods

Subjects who solely had a lifetime wish for amputation where placed in the amputation-group, whereas subjects with another wish for disability than solely amputation, were placed in the paralyzation-group. The small sizes of groups limited statistical analysis. We therefore describe the results qualitatively.

Upon qualitatively screening the results, large differences were seen in SDS scores between amputated versus non amputated subjects. Therefore statistics were performed to compare these scores. The difference was analyzed using the Mann-Whitney U-test, a non parametric test. Predictive Analytic Software (PWAS) for Windows 18.0 (SPSS Inc, Chicago Illinois) was used to perform these statistical analyses.

## Results

Fifty-four BIID subjects completed the survey and indicated that the main rationale for their desire for body modification is *to feel complete* or *to feel satisfied inside* [1]. Sexual arousal concerning their BIID (i.e. being aroused when seeing someone disabled resembling their BIID or when imagining themselves being disabled) was present in almost half of the subjects, but was never the primary rationale for their desired body modification. 79.6% were males, 96.3% were of Caucasian origin, 64.8% had a university degree, and age range was 18–76 years.

Amputation of one or more limbs was preferred by 30 (55.6%) *“I can feel exactly the line where my leg should end and my stump should begin. Sometimes this line hurts or feels numb.”* Twenty-four (44.4%) wished to be disabled in another way than limb amputation. Of those most (23/24) wished to have a form of paralysis and one preferred to have club-feet (Table 1). Upon qualitatively screening the results the two groups did not indicated an important difference on any item. Fifteen subjects (27.8%) described their preferred body part had changed overtime: (e.g. 5 went from leg amputation to spinal cord paralysation).

**Table 1:** BIID manifestations as self-reported in questionnaires in BIID individuals.

	Amputation (n = 30)	Paralysation (n = 24)	Total (n = 54)
**Age of onset (mean – range)**	7.0 (3–12)	6.3 (3–15)	6.7 (3–15)
**Females (biological sex) (n – (%))**	3 (10.0)	7 (29.2)	10 (18.5)
**Site (n – (%))** *	Left 11 (36.7)	Left 0 (0.0)	Left 11 (20.4)
	Right 9 (30.0)	Right 0 (0.0)	Right 9 (16.7)
	Bilateral 10 (33.3)	Bilateral 24 (100.0)	Bilateral 34 (63.0)
**Change site over time (n – (%))**	10 (33.3)	5 (20.8)	15 (27.8)
**Presence (n – (%))**			
-Always	14 (46.7)	8 (33.3)	22 (40.7)
-Sometimes limited	13 (43.3)	13 (54.2)	26 (48.1)
-Sometimes absent	3 (10.0)	3 (12.5)	6 (11.1)
**Body modification (n – (%))**			
Ever thought of it	27 (90.0)	20 (83.3)	47 (87.0)
Ever tried myself	10 (33.3)	6 (25.0)	16 (29.6)
Consulted physician	12 (40.0)	4 (16.7)	16 (29.6)
Modification is performed	7 (23.3)	-	7 (13.0)

* 3 left under knee amp; 8 left above knee amp; 7 right above knee amp; 1 right under knee amp; 6 bilateral above knee amp; 2 bilateral under knee amp; 1 right above elbow amp; 1 bilateral above elbow amp; 1 tetra amputation; 18 lower back paralysation; 1 spastic paraperesis of legs; 1 lower back paralysation and left below elbow amp; 1 paralysis starting at thighs; 2 partial lower back paralysation; 1 clubfeet.

Physical comorbidity appeared to be infrequent (Table 2). If BIID individuals had a unilateral modification preference (n = 20), 70.0% expressed a wish for amputation on the non-dominant side of their body. Medical problems concerning the affected body part (i.e. the part of the body they wish to be either amputated or paralyzed) were all reported to develop after onset of the BIID. Some (such as muscle weakness) were due to manifest avoidance of use of the affected body part. Individuals reporting their body part to feel different, often explain that their limb feels alien: *“My limbs do not feel like they belong to me, and should not be there”*.

**Table 2:** Social aspects of 54 BIID individuals as self-reported in questionnaires.

	Amputation (n = 30)		Paralysation (n = 24)		Total (n = 54)	
**In a relationship with significant other (n – (%))**	23 (76.6)		10 (41.7)		33 (61.1)	
**Sexual orientation (n – (%))**						
-Heterosexual	17 (56.7)		13 (54.2)		30 (55.6)	
-Homosexual	8 (26.7)		7 (29.2)		15 (27.8)	
-Bisexual	5 (16.7)		4 (16.7)		9 (16.7)	
**Specific sexual desires (n – (%))**						
Aroused when seeing someone disabled resembling my BIID	14 (46.7)		11 (45.8)		25 (46.3)	
Aroused when imagining myself being disabled	15 (50.0)		9 (30.0)		24 (44.4)	
Aroused when dressing like the other gender	2 (6.7)		1 (4.2)		3 (5.6)	
**Disclose BIID (n – (%))**						
To partner (in case of having one)	18 (72.0)		9 (60.0)		27 (67.5)	
To close friends (in case of having close friends)	15 (50.0)		16 (66.7)		31 (57.4)	
To close family (in case of having family)	10 (33.3)		6 (25.0)		16 (29.6)	
**Sheehan Disability Scale (without/with modification)**	n = 23	n = 7	n = 24	n = 0	n = 47	n = 7
BIID disrupts work (mean – range)	6.6 (1–10)	1.6 (1–3)	5.7 (1–10)	-	6.1 (1–10)^A^	1.6 (1–3)^A^
BIID disrupts social life	6.0 (1–10)	1.3 (1–2)	5.8 (1–10)	-	5.9 (1–10)^B^	1.3 (1–2)^B^
BIID disrupts family life	5.8 (1–10)	1.9 (1–3)	4.9 (1–10)	-	5.4 (1–10)^C^	1.9 (1–3)^C^
BIID disrupts personal happiness	8.7 (3–10)	1.6 (1–4)	7.8 (1–10)	-	8.2 (1–10)^D^	1.6 (1–4)^D^
**Treatment (n – (%))**						
Professional help sought	15 (50.0)		9 (37.5)		24 (44.4)	
Psychiatric medication taken	9 (30.0)		6 (25.0)		15 (27.8)	
Psychological or behavioural therapy	10 (33.3)		6 (25.0)		16 (29.9)	
Surgical treatment	7 (23.3)		0 (0.0)		7 (13.0)	
Medication was helpful	3 (33.3)		2 (33.3)		5 (33.3)	
Therapy was helpful	4 (40.0)		2 (28.6)		6 (35.3)	
Surgery was helpful	7 (100.0)		-		7 (100.0)	

A (Mann-Whitney U = 33.5, p<0.001).

B (Mann-Whitney U = 27.0, p<0.001).

C (Mann-Whitney U = 61.0, p<0.01).

D (Mann-Whitney U = 7.0, p<0.001).

Diagnoses of lifetime psychiatric co-morbidity (Table 3) were based on self-report of diagnoses made by participants' therapists. Current psychiatric disorders were also scored positive in the M.I.N.I. screen, which reinforced the likelihood of their presence. One subject was diagnosed with schizophrenia. Her BIID feelings were present as long as she could remember, from the age of 5, while her hallucinations started later on. The hallucinations involved ‘her name being called’ and visual hallucinations such as ‘the room moving when turning her head’. Her hallucinations diminished in strength following antipsychotic medication, but had no influence at all on her BIID feelings. In addition, her BIID feelings were always present, whereas her hallucinations tended to come in waves. Moreover, her motivation for paralyzation was her wish *to feel complete*, and not *a punishment of God*, of *getting rid of the Devil* as seen in self-performed amputations in schizophrenics [5]. We concluded that her BIID thoughts differed enough from her hallucinations that her wish for amputation was not psychotic. None of the other subjects reported a psychotic psychiatric diagnosis and neither scored positive on the M.I.N.I. screen for psychotic symptoms. Depressive and anxiety symptoms were reported somewhat higher than in the general population.

**Table 3:** Somatic and psychiatric aspects of 54 BIID individuals as self-reported in questionnaires.

	Amputation (n = 30)	Paralysation (n = 24)	Total (n = 54)
**Height in cm (mean – range)**	179.8 (167–198)	178.8 (163–196)	179.3 (163–198)
**Weight in kg (mean – range)**	83.6 (59–122)	77.8 (54–122)	81.0 (54–122)
**Body mass index (mean – range)**	25.9 (19–37)	24.2 (16–40)	25.2 (16–40)
**Head Circumference (mean – range)**	57.0 (50–61)	57.6 (55–60)	57.3 (50–61)
**Handedness (n – (%))**			
-Right handed	22 (73.3)	22 (91.7)	44 (81.5)
-Left handed	6 (20.0)	2 (8.3)	8 (14.8)
-Ambidexter	2 (6.7)	0 (0.0)	2 (3.7)
**Abnormalities of the affected body part(s)** *			
Feels different inside	12 (40.0)	12 (50.0)	24 (44.4)
Feels different if someone touches	12 (40.0)	10 (41.7)	22 (40.7)
Feels different when temperature changes	3 (10.0)	8 (33.3)	11 (20.4)
Medical problems	7 (23.3)	5 (20.8)	12 (22.2)^A^
**Neurological problems (n – (%))**	3 (10.0)	4 (16.7)	7 (13.0)^B^
**Cardiovascular abnormalities (n – (%))**	1 (3.3)	1 (3.3)	2 (3.7)^C^
**Pulmonary abnormalities (n – (%))**	4 (13.3)	3 (12.5)	7 (13.0)^D^
**Gastrointestinal abnormalities (n – (%))**	4 (13.3)	1 (4.2)	5 (9.3)^E^
**Other abnormalities (n – (%))**	5 (16.7)	5 (20.8)	10 (18.5)^F^
**Psychiatric co-morbidity (lifetime) (n – (%))**	7 (23.3)	11 (45.8)	18 (33.3)
Mood disorder	6 (20.0)	7 (29.2)	13 (24.1)
Anxiety disorder	1 (3.3)	1 (4.2)	2 (3.7)
Psychotic disorder	0 (0.0)	1 (4.2)	1 (1.9)
Eating disorder	0 (0.0)	2 (8.3)	2 (3.7)
**Back Anxiety Inventory (mean – range)**	14.7 (6–35)	15.7 (6–35)	15.1 (6–35)
**Beck Depression Inventory (mean – range)**	11.7 (0–40)	14.3 (0–42)	12.9 (0–42)
**Adapted version of the Y-BOCS (mean – range)**	13.7 (8–18)	12.8 (10–16)	13.3 (8–18)

* Part(s) of the body BIID individuals wish to be either amputated or paralysed.

A 4x fractures to arms/legs; 1x spinal fracture; 1x restless toes; 1x spinal compression; 1x knee injury; 1x morton's neuroma; 2x muscle problems; 1x diabetic neuropathy.

B 5x lumbar hernia; 1x muscle spasms; 1x fibromyalgia;

C 1x heart attack; 1x valve problems;

D 1x asthma; 2x bronchitis; 4x pneumonia;

E 1x stomach problems; 1x pancreatitis; 1x cholangiolithiasis; 1x colitis; 1x appendicitis.

F 1x lower back pain 1x lipomata; 1x spinal fracture; 1x hypothyroidism; 1x scoliosis; 3x diabetes; 1x immune depression; 1x renal colic; 2x intersex condition (one ambiguous genitalia, surgically corrected; other male genitalia but female identity).

The social impact of having BIID was enormous (Table 2). *“BIID occupies every waking moment of my life, and even keeps me awake at night. Insomnia is severe most nights”*. Severity of symptoms such as the obsession with their limbs was severe (13.2 out of 20 on the adapted version of the Y-BOCS). Psychotherapy was often supportive, but did not help diminishing BIID symptoms: “*While psychotherapy did not help BIID directly, it did help understanding my relationship to BIID.*” Antidepressants were felt helpful to reduce depressive symptoms related to BIID, but antipsychotics were not. Actual amputation of the limb was effective in all 7 cases who had surgical treatment. *“I'm wondering if I am eligible to participate in this study, because since my amputation I do not have BIID feelings anymore”*. Comparisons on the SDS of subjects with and without amputation were significant in all items, suggesting less disability after amputation (Table 2).

## Discussion

A large group of BIID individuals (n = 54) was phenotyped using a questionnaire. BIID has an onset in early childhood; 80% are men. Main rationale given for their desire for body modification is to feel complete or to feel satisfied inside, sexual motives are often secondary. Prevalence rates of homosexual and bisexual orientation are high. Somatic and severe psychiatric co-morbidity is unusual, but depressive symptoms and mood disorders can be present, possibly secondary to the enormous distress BIID puts upon a person. BIID influences lives of affected subjects in all facets in an extreme way. Subjects that underwent amputation score significantly lower on a disability scale than BIID subjects who did not undergo body modification, suggesting that surgery does offer benefits to subjects.

Three observational studies have described BIID individuals before [1], [15], [16]. This report extended those studies by using a larger group of participants, recruiting individuals with an identity disorder (instead of a wish for amputation) and including the description of the paralyzation variant. Our results seem to be largely in keeping with those reported before.

Concurring with previous literature, we also find that the level of distress in BIID subjects is high [1]. Obsessions with BIID are present every day, many individuals spent time pretending, using crutches, bandage their limbs or using a wheelchair. “*I am using a wheelchair “full time” when I'm in public. I walk at home. This is the only way how to remain somewhat functional.”* The thoughts and activities around BIID disrupt social life, work, and family life. BIID individuals disclose their BIID to their family and friends in just half of the cases.

Subjects who actually had performed amputation scored significantly lower on the *Sheehan Disability Scale* compared to those who had not. BIID individuals prefer being in harmony with one's identity, even if it results in physical disability. Surgery appears to result in permanent remission of BIID and in impressive improvement of quality of life, but conflicts with ethical standards of physicians indicating not to amputate healthy limbs [25], [26].

Since there are no clear differences in any other parameter between the amputation and paralyzation BIID variants, we consider these as the same condition. We hypothesize that amputation of the body part affected in the paralyzation variant would usually lead to incompatibility with life if it would be amputated and therefore people (unconsciously) prefer to be paralyzed. Alternatively individuals with the paralyzation variant may specifically seek to be paralyzed as such.

In the present study the main reasons reported for body modification in all subjects were to feel whole, complete, set right again or to feel satisfied inside, none of the subjects had primary sexual motives. However 25 (46.3%) subjects felt sexually aroused when seeing someone disabled resembling their BIID and 24 (44.4%) felt sexually aroused when imagining themselves being disabled. Possibly the sexual component in BIID is often one of feeling sexually more comfortable with one's body [1]. *“I maybe am more comfortable sexually with myself and others as an amputee, because I would be a complete person.”*

Physical co-morbidity reported both here and in literature is infrequent, and prevalence is probably not different to that in the general population. Detailed comparisons are hampered by the widespread geographical distribution and age range of participants. Possibly, the occurrence of a lumbar hernia (n = 5 in present study and n = 4 in Blanke et al. [15]) may be higher. In all, the BIID onset preceded the hernia manifestations. Two BIID individuals with the paralyzation form from in the present study and one with the amputation form reported by First et al. stated to have an intersex condition (see Table 3) [1]. These rates are substantially higher than in the general population [27] and therefore might suggest a common pathway in developing identity disorders [2]. However there can be a significant ascertainment bias, so it still remains uncertain at present whether there is a true relation between intersexuality and BIID.

Psychiatric co-morbidity in present study and literature shows no obvious difference compared to the general population, except for an increase in depressive symptoms and mood disorders in present study and also in literature [1], [15]. We suggest these symptoms to be secondary to BIID due to the high distress level, and not to represent a separate manifestation. One of the BIID individuals was schizophrenic, however her BIID thoughts were not considered as part of a psychosis. On the other hand, amputation due to psychosis is known to occur but is not considered to be BIID since the motivation for amputation is often delusional like “performing mission for God” or “Rid herself of a devil that had entered hand and made her do bad things” [5].

The present study shows high rates of bisexual and homosexual orientation in BIID individuals, as reported by most [1], [28], [29] but not all others [15]. One might speculate that the presence of a less prevalent sexual orientation makes a person more open to speak about their BIID identity.

The aetiology of BIID remains unclear. Congenital abnormal body representation in the brain has been proposed [3], [6], [7], [10]. Time of onset (usually from as early as BIID individuals can remember), similarities with somatophrenia, and persisting exactness of line of wished amputation are arguments for such a deficit. The preliminary finding of absence of activity in the right superior parietal lobule when stimulating the affected body area may supports this [10], [30]. Arguing against is the change in affected body part and intensity over time in some BIID individuals, but this does not exclude a neurological cause with certainty. We hypothesize a multigenic origin of BIID and have recently initiated molecular studies using next generation sequencing techniques.

### Limitations

Strength of the study is the presentation of somatic, psychiatric, social and BIID characteristic data of a large group of BIID individuals, including the paralyzation variant. Some limitations must be noted. The major limitation of the study is the lack of in person structured interviews and physical examinations of the participants. BIID is a rare and extremely secretive condition, which forms a major obstacle for in person or phone evaluations of a large group of affected individuals. To generate a sample of sufficient size, we decided to restrict communication through the internet. Indeed, it has been suggested that this is unavoidable in studies of such rare disorders [31]. Study participants had to answer questions written in English while this was not always their mother tongue. We allowed them to answer the open questions in their mother tongue however. Moreover, as BIID is rare and highly secretive, we cannot exclude with certainty that there is no overlap between cases reported in literature and the present study participants. For the paralyzation variant we do know these have not been reported, and results in this group and in the amputation variant are very similar which adds to the reliability of the results. Lastly, due to a limited sample size and widespread origin of the participants, results should be generalized only with caution.

### Conclusion

BIID is a rare, infrequently studied and highly secretive condition in which a mismatch between mental body image and the physical body influences lives of affected persons in an extreme way. BIID results in an intense desire to amputate a major limb or severe the spinal cord in order to become paralyzed and may lead individuals to self-inflicted mutations. For affected individuals, BIID desires are essential to life and not the result of major somatic or psychiatric morbidity. Further research is warranted to reveal the aetiology of this condition. Physicians need to be aware of BIID when meeting someone with a wish for unusual body modifications. Careful discussions of this desire are essential. Next to surgery there is no effective management strategy at present but the sheer acknowledgment of and respect for the desires of BIID individuals may decrease the huge burden of BIID on their lives.

## Supporting Information

Figure S1 **Questionnaire Body Integrity Identity Disorder.**
(PDF)

Figure S2 **Adapted version of the Yale Brown Obsessive Compulsive Scale.**
(PDF)
