# Supplementary material for: Body Integrity Identity Disorder
Source: PLoS One. 2012 Apr 13;7(4):e34702. doi: 10.1371/journal.pone.0034702 (PMC3326051; doi:10.1371/journal.pone.0034702)
Supplement: Figure S1 — Questionnaire Body Integrity Identity Disorder. (PDF) [file pone.0034702.s001.pdf]

QUESTIONNAIRE BODY INDENTITY INTEGRITY DISORDER

Dear sir/madam,

You have been asked to participate in a research of the AMC to BIID. This part of the research consists of a questionnaire; which we will use to find characteristics occurring in individuals with BIID. Some questions will be general; other questions will be BIID related.

BIID is a term that covers several conditions in which people feel their body-image does not match with their body shape. When we use the term "BIID" or "BIID feelings" here we mean to indicate all these different forms of the condition. For example, some people would like to have their leg to be amputated under their knee, whereas others prefer to resemble someone who is paralysed.

It will take about 60 minutes to fill in the questionnaire. The questions are in English; however you may answer the questions in English, French, German or Dutch.

If you have any questions; please do not hesitate to contact us.

Thank you for your help,

Rianne Blom MSc, Prof. Damiaan Denys, and Prof. Raoul Hennekam

QUESTIONNAIRE BODY INTEGRITY IDENTIY DISORDER

GENERAL

Date: …

Research number: …

Year of birth: …

Ethnic background: Caucasian (European) / Caucasian (American) / Asian / Hispanic / African

Sex: male (XY) / female (XX)

Gender: male / female

Eye color: green / blue / brown / grey

Hair color (natural): blond / dark blond / brown / black / red

Length: … cm or … feet

Weight: … kg or … lb

Head circumference: … cm or … inches

Education (graduated): primary school / secondary school / higher education / university degree

Sexual orientation: bisexual / heterosexual / homosexual

Transsexual: yes / no

Handedness: right-handed / left-handed

Religion: none / Buddhist / Catholic / Hindu / Jewish / Protestant / Muslim / other

Marital status: single / divorced / married / in a relationship / widowed

Do you have children: yes / no

Number of tattoos: 0 1 2 3 4 5<

Number of piercings: 0 1 2 3 4 5<

BIID SPECIFIC

ONSET

At what age did your first BIID feelings start? … year

Who has identified you with BIID? self / general practitioner / psychiatrist / other physician*

At what age have you been identified with BIID? … year

Did you experience a specific trigger at the moment you first BIID feelings started? yes / no

If yes, could you please describe this/these triggers?

….

MEDICAL

Have you ever medical problems with BIID affected body part(s) such as ulcers, operations, infections, fractures, diseases? yes / no

If yes, can you please describe this / these problems?

…..

Does your BIID body part(s) feels inside different than your non BIID body part(s)? yes / no

If someone touches your BIID body part(s), does that feel different than when someone touches other body parts? yes / no

If yes, can you describe the difference?

…

If the temperature changes heavily, does your BIID body part reacted different than your other body parts?

yes / no

If yes, can you describe the difference?

…

BIID DESCRIPTION

How would you yourself describe your BIID? Please be as exact as possible.

…..

Below we have indicated several examples of BIID feelings. Can you please indicate to which you identify yourself most? It is possible to mark more than one condition.

Left upper leg amputation / Right upper leg amputation /

Left under knee amputation / Right under knee amputation /

Left foot amputation / Right foot amputation /

Left under arm amputation / Right under arm amputation /

Left upper arm amputation / Right upper arm amputation /

Left hand amputation / Right hand amputation /

Paralysed at cervical level / Paralysed at lower back level /

Partial-paralysed at cervical level / Partial-paralysed at lower back level /

Other

If other, please indicate how you would prefer to describe your BIID?

.....

Has your BIID changed over time? yes/no

If so can you please describe your BIID just after the onset and how it is now?

.....

Did your BIID concern different body parts over time? yes / no

If no, what did change?

…

Or did the BIID body part remain stable over time? yes / no

What was the course of your BIID?

Present all the time /sometimes fully present and sometimes present only in a limited way / sometimes fully present and sometimes complete absent

What was the start of your BIID?

Acute / Slowly

If slowly, over what period of time did it start?

…

Do you yourself have an explanation why you experience BIID?

.....

Some BIID people have mentioned in the past several reasons why they would like their body to be different as it was. Can you please indicate which description would fit you best?

**Reason 1: "Because of the attention it draws"**	yes / no
**Reason 2: "In order to be disabled and have others help me"**	yes / no
**Reason 3: "In order to feel whole, complete, set right again"**	yes / no
**Reason 4: "In order to feel sexually aroused or excited myself"**	yes / no
**Reason 5: "In order to feel satisfied inside"**	yes / no
**Reason 6: "Process of body-modification is the main focus"**	yes / no

If none of the reasons mentioned above fits you well or describes it completely, can you please describe which reason is for you important?

...

If more than one reason would fit you, can you please indicate a row of order of importance to you of the various reasons?

…

What is the most important reason?

Reason 1: “Because of the attention it draws”

Reason 2: “In order to be disabled and have others help me”

Reason 3: “In order to feel whole, complete, set right again”

Reason 4: “In order to feel sexually aroused or excited myself”

Reason 5: “In order to feel satisfied inside”

Reason 6: “Process of body-modification is the main focus”

Other:

What is the second most important reason?

Reason 1: “Because of the attention it draws”

Reason 2: “In order to be disabled and have others help me”

Reason 3: “In order to feel whole, complete, set right again”

Reason 4: “In order to feel sexually aroused or excited myself”

Reason 5: “In order to feel satisfied inside”

Reason 6: “Process of body-modification is the main focus”

None

Other:

What is the third most important reason?

Reason 1: “Because of the attention it draws”

Reason 2: “In order to be disabled and have others help me”

Reason 3: “In order to feel whole, complete, set right again”

Reason 4: “In order to feel sexually aroused or excited myself”

Reason 5: “In order to feel satisfied inside”

Reason 6: “Process of body-modification is the main focus”

None

Other:

MODIFICATION

Have you ever thought of modifying your body yourself? yes / no

Have you ever tried to modify your body yourself? yes / no

If yes, can you please describe how you proceeded and whether you succeeded?

Have you ever consulted a professional (like a surgeon) to modify your body? yes / no

If yes, did the professional help you to get the modification done? yes / no

If yes, can you please describe what has been done to your body?

.....

SEXUAL

Some people sometimes experience specific sexual desires. Can you please indicate whether one or more is true for you?

"I feel sexually aroused when thinking of someone disabled resembling my BIID" yes / no

"I feel sexually aroused when seeing someone disabled resembling my BIID" yes / no

"I feel sexually aroused when I image myself being disabled" yes / no

"I feel sexually aroused when I act like I'm disabled" yes / no

"I feel sexually aroused when dressing like the other gender" yes / no

"I feel sexually aroused when seeing specific non-living objects" yes / no

BIID TREATMENT

Have you ever sought professional help for you BIID feelings? yes / no

Yes, I have contacted my: general physician / surgeon / psychiatrist / neurologist / other / .....

Have you ever been given any of the following treatments because of BIID?

**Antidepressants**	yes / no	**Was it helpful?**	yes / no
**Antipsychotics**	yes / no	**Was it helpful?**	yes / no

**Behavioural therapy**	yes / no	**Was it helpful?**	yes / no
**Psychotherapy**	yes / no	**Was it helpful?**	yes / no
**Surgery**	yes / no	**Was it helpful?**	yes / no

MEDICAL GENERAL

Have you ever suffered from a physical illness? yes / no

If yes, can you please describe this illness?

…..

Are you suffering from a physical illness at present? yes / no

If yes can you please describe this illness?

…..

Do you have a chronic medical disorder? (Such as diabetics or hypertension) yes / no

If yes, can you please describe this disorder?

…..

Did you ever have surgery? yes / no

If yes, please describe this/these operation(s) and your age at surgery

…..

Did you have a serious head injury as a child? yes / no

If yes, can you please describe this injury and your age when you had this trauma?

.....

Do you have a psychiatric illness at present? yes / no

If yes, please describe this illness?

.....

Have you had a psychiatric illness in the past? yes / no

If yes, please describe this illness and at which age?

.....

GENERAL TREATMENT

Are you using any medication at the moment? yes / no

Please describe which medication(s) you take

1. Name: ______________________ Dose: ____________ Using since: ___________

2. Name: ______________________ Dose: ____________ Using since: ___________

3. Name: ______________________ Dose: ____________ Using since: ___________

FAMILY

Do close friends of you know of your BIID? yes / no / I have no close friends

Does your partner know of your BIID? yes / no / I have no partner

Does your family know of your BIID? yes / no / I have no family

Has someone of your family BIID as well? yes / no / not to my knowledge

If yes, please indicate who in your family is having BIID as well?

father / mother / son / daughter / grandfather / grandmother / cousin / niece / nephew / aunt / uncle / brother / sister/ other, ...
