# Supplementary material for: Body Integrity Identity Disorder
Source: PLoS One. 2012 Apr 13;7(4):e34702. doi: 10.1371/journal.pone.0034702 (PMC3326051; doi:10.1371/journal.pone.0034702)
Supplement: Figure S2 — Adapted version of the Yale Brown Obsessive Compulsive Scale. (PDF) [file pone.0034702.s002.pdf]

Box 1: Adapted version of the Yale Brown Obsessive Compulsive Scale

YBOCS

There will follow some questions concerning the time that you are busy with your BIID and how much this interfere with your life. Examples of your ‘being busy with your BIID’ are: thinking of your BIID, fantasizing how it would be have a modified body, binding your legs, sitting in a wheelchair, speaking on the internet with peers, regarding images of disabled people on the internet etc.

1. How much of your time is occupied by your thoughts and activities around your BIID?

0. None

1. Mild (less than 1 hour a day)

2. Moderate (1 to 3 hours a day)

3. Severe (3 to 8 hours a day)

4. Extreme (more than 8 hours a day)

2. How much do your thoughts and activities around your BIID interfere with your social or work functioning?

0. None

1. Mild, slight interference with social or occupational activities, but overall performance not impaired

2. Moderate, definite interference with social or occupational performance, but still manageable

3. Severe, causes substantial impairment in social or occupational performance

4. Extreme, incapacitating

3. How much distress do your thoughts and activities around your BIID cause you?

0. None

1. Mild, not too disturbing

2. Moderate, disturbing but still manageable

3. Severe, very disturbing

4. Extreme, near constant and disabling distress

4. How much of an effort do you make to resist the thoughts and activities around your BIID?

0. I make an effort to always resist, or thoughts and activities are so minimal I do need to actively resist

1. I try to resist most of the time

2. I make some effort to resist

3. I rarely make an effort to resist

4. I never make an effort to resist

5. How much control do you have of your thoughts and activities around your BIID?

0. Complete control

1. Much control, usually able to stop or divert thoughts and activities

2. Moderate control, sometimes able to stop or divert thoughts and activities

3. Little control, rarely successful in stopping or dismissing thoughts and activities

4. No control, thoughts and activities experienced as completely involuntary
